# Dysfunctional High-Density Lipoprotein: An Innovative Target for Proteomics and Lipidomics

**DOI:** 10.1155/2015/296417

**Published:** 2015-11-08

**Authors:** Juan Salazar, Luis Carlos Olivar, Eduardo Ramos, Mervin Chávez-Castillo, Joselyn Rojas, Valmore Bermúdez

**Affiliations:** Endocrine-Metabolic Research Center, “Dr. Félix Gómez,” Faculty of Medicine, University of Zulia, Zulia State, Maracaibo 4004, Venezuela

## Abstract

High-Density Lipoprotein-Cholesterol (HDL-C) is regarded as an important protective factor against cardiovascular disease, with abundant evidence of an inverse relationship between its serum levels and risk of cardiovascular disease, as well as various antiatherogenic, antioxidant, and anti-inflammatory properties. Nevertheless, observations of hereditary syndromes featuring scant HDL-C concentration in absence of premature atherosclerotic disease suggest HDL-C levels may not be the best predictor of cardiovascular disease. Indeed, the beneficial effects of HDL may not depend solely on their concentration, but also on their quality. Distinct subfractions of this lipoprotein appear to be constituted by specific protein-lipid conglomerates necessary for different physiologic and pathophysiologic functions. However, in a chronic inflammatory microenvironment, diverse components of the HDL proteome and lipid core suffer alterations, which propel a shift towards a dysfunctional state, where HDL-C becomes proatherogenic, prooxidant, and proinflammatory. This heterogeneity highlights the need for further specialized molecular studies in this aspect, in order to achieve a better understanding of this dysfunctional state; with an emphasis on the potential role for proteomics and lipidomics as valuable methods in the search of novel therapeutic approaches for cardiovascular disease.

## 1. Introduction

Cardiovascular disease (CVD) constitutes the worldwide leading cause of morbidity and mortality, unequivocally representing an alarming public health problem [1]. Various conditions, termed* risk factors*, are associated with the onset and progression of CVD, including hypertension, obesity, and dyslipidemias [2, 3], framed in a lifestyle characterized by several deleterious habits, such as fat- and carbohydrate-rich diets, smoking, and physical inactivity [4], augmenting the probability of suffering CVD. Among these factors, dyslipidemias emerge as some of the most significant etiopathogenic components given their close link to the atherosclerotic process, being considered the main therapeutic target in the management of CVD [5].

Dyslipidemias are disorders of lipid metabolism, manifested as an abnormal increase or decrease of serum lipids. Diverse reports support a beneficial role for High-Density Lipoproteins (HDL) in regard to cardiovascular health, boasting powerful antiatherogenic properties [6, 7]. These are mediated by a phenomenon known as reverse cholesterol transport (RCT), through which cholesterol molecules are carried from cells in vascular walls and other organs to the liver, where they may be reutilized or excreted [8]. Likewise, other antiatherogenic attributes comprise inhibition of monocyte adherence to the endothelium and monocytes migration to the arterial intima, prevention of vascular thrombosis, stimulation of endothelial repair, and many others [9]. Epidemiologic studies like the* Cooperative Lipoprotein Phenotyping Study* [10] and the* Framingham Heart Study* [11] have identified HDL cholesterol (HDL-C) to be intimately related to the atherosclerotic process, where serum HDL-C concentrations have been demonstrated to have an inverse relationship with CVD incidence. Indeed, in the latter, a 10 mg/dL increase in HDL-C concentration has been associated with not only lower cardiovascular mortality, but also decreased all-cause mortality [12].

In spite of these findings, there is conflictive evidence regarding the “protective” role of HDL-C in cardiovascular health: certain hereditary syndromes featuring scant HDL-C levels have been shown not to display early atherosclerosis [13–15]; and very high levels of this lipoprotein do not appear to grant additional benefits [16]. Indeed, current knowledge suggests the biologic activity of HDL may depend on both their quantity and their quality, as alterations in various structural components lead to a state of dysfunction independently of their serum concentration [17]. Given the epidemic CVD has grown into, and the rising scientific interest in HDL as a therapeutic target, it is necessary to understand the molecular aspects underlying their dysfunction and possible approaches for their evaluation.

## 2. Metabolism of High-Density Lipoprotein-Cholesterol

Structurally, HDL units may be described as macromolecular pseudomicellar complexes, characterized by exhibiting the greatest density (1.063–1.21 g/mL) and smallest size (4–13 nm) among all lipoproteins [18]. Multiple HDL molecules may associate, forming a completely hydrophobic nucleus rich in cholesteryl esters (CE), covered by a layer of amphipathic lipids and proteins. HDL molecules also possess important quantities of CE on the lipoprotein surfaces employed for interaction with the enzymes intervening in their metabolism [19]. These mediators include hepatic lipase, cholesteryl ester transfer protein (CETP), Phospholipid Transfer Protein (PLTP), and Lecithin-Cholesterol Acyltransferase (LCAT) [20–22].

**Figure 1:**
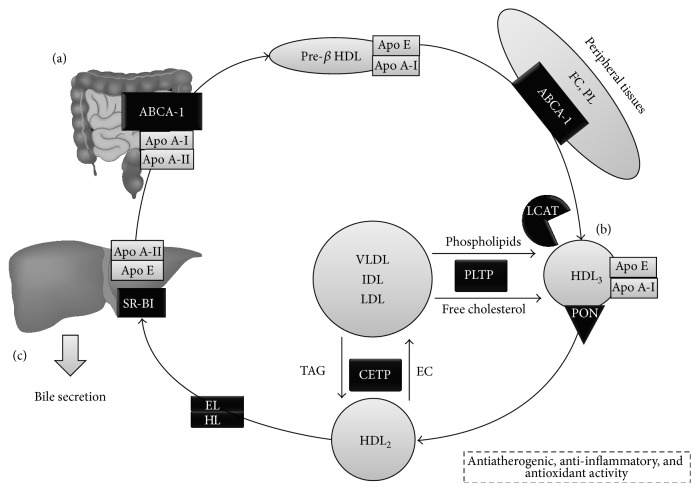
HDL metabolism and main components implicated in their antiatherogenic-anti-inflammatory functions. HDL metabolism consists of 3 phases. (a) Synthesis occurs in the liver and intestine, originating discoid or pre-*β* HDL. This subpopulation initiates RCT in peripheral tissues, mediated by Apo A-I binding to ABCA-1 and LCAT, resulting in HDL rich in cholesteryl esters. (b) HDL_3_ are the first to form, which continue cholesterol capture in various tissues. Likewise, CETP transfers TAG to HDL, whereas PLTP mediates transfer of phospholipids and free cholesterol, increasing the size of the particles, yielding HDL_2_. (c) Finally, HDL undergos exclusion through SR-B1 in hepatocytes, for either biliary secretion or formation of new lipoproteins. FC: free cholesterol; PL: phospholipids; ABCA-1: ATP-binding cassette transporter A-1; LCAT: Lecithin-Cholesterol Acyltransferase; PON: Paraoxonase; CETP: cholesteryl ester transfer protein; HL: hepatic lipase; EL: endothelial lipase; HDL: High-Density Lipoprotein; SR-B1: Scavenger Receptor B1.

Metabolism of HDL is organized in three fundamental stages: formation, maturation, and exclusion (Figure 1). Initially, HDL are synthesized and secreted from the liver and intestine as nascent pre-*β*-1 HDL or discoid HDL particles, conformed predominantly by apolipoprotein A-I (Apo A-I) and phospholipids. These particles then arrive at peripheral tissues and remove free cholesterol (FC) as part of RCT [23]. Besides, these lipid-poor, Apo A-I-rich, nascent HDL particles are capable of removing FC through the ATP-binding cassette transporter A-1 (ABCA-1), which transfers these lipids towards the interior of the pre-*β*-1 lipoprotein [24]. Finally, LCAT acts by esterifying FC present in these pre-*β*-1 lipoproteins, originating CE-rich, mature HDL [25], while ATP-binding cassette transporter G-1 (ABCG-1) mediates cholesterol efflux to these mature HDLs [26].

In this stage, CE now occupy and contribute to the lipid core of the micelle, marking the transition from nascent HDL into small HDL_3_, which can bind to their mature, spherical counterparts in the liver and intestines. Due to their low cholesterol content, small HDL_3_ are able to continue collecting excess cholesterol in cell membranes [26]. Once no substrates for LCAT remain, HDL_3_ begin accepting phospholipids, cholesterol, and apoproteins from other lipoproteins, derived from the activity of lipoprotein lipase on chylomicrons and very low-density lipoprotein (VLDL). Indeed, PLTP mediates the transfer of excess phospholipids from the surface of TAG-rich lipoproteins to HDL, facilitating the formation of lipoprotein remnants and contributing to HDL maturation, which further increase in size and become phospholipid-rich HDL_2_ [27]. PLTP also drives a phenomenon termed conversion [28], which involves fusion of several HDL particles, generating larger HDL with concomitant production of small lipid-poor Apo A-I/phospholipid complexes [29]. Finally, PLTP is also an essential component in RCT, not only by facilitating cholesterol efflux from peripheral tissues [30], but also by yielding pre-*β*2 HDL, lipid-poor particles during conversion, which act as initial acceptors of this cholesterol efflux [31], and by stabilizing ABCA-1, thus promoting phospholipid and cholesterol traffic by this intermediary [32].

Phospholipid-rich HDL_2_ are also LCAT substrates, allowing removal of FC and phosphatidylcholine (PC) excess accumulated in cell surfaces; this results in an increase of lipoprotein volume, reflecting growing quantities of CE in their core [33]. In addition, CETP also catalyzes the exchange of CE for triacylglycerides (TAG) from HDL_2_ to VLDL [33, 34], which is essential for maintaining a hydrophobic environment in the core [35]. CETP seems to achieve this through three powerful mechanisms [7]. Firstly, CETP can act freely as a lipid transporter between lipoproteins. It is also involved in the assembly of ternary donor-acceptor complexes with lipoproteins. Lastly, CETP also participates in the arrangement of other distinct ternary complexes, featuring CETP dimers which aid in the formation of binding sites for lipoproteins [34]. These TAG-rich HDL are now optimal targets for hepatic and endothelial lipases, which hydrolyze TAG and phospholipids, thus favoring HDL catabolism, retransitioning from HDL_2_ to HDL_3_ and pre-*β* HDL [36].

Lastly, during exclusion, HDLs release cholesterol directly to the liver and steroidogenic tissues through selective HDL capture mediated by Scavenger Receptor class B type I (SR-BI), releasing particles integrated into its structure without undergoing complete degradation [35]. This process delivers cholesterol for synthesis and secretion of new lipoproteins and biliary excretion in the liver, as well as synthesis of steroid hormones in steroidogenic tissues [37]. Ulterior catabolism of HDL particles occurs mainly in the liver and kidney, finalizing their life cycle [38].

Another pathway involved in HDL metabolism is endocytosis, facilitated by SR-BI in peripheral tissues, followed by resecretion—now rich in cholesterol—to the vascular medium, contributing to cholesterol efflux [39, 40]. This mechanism is very important in the origin of foamy cells in atheromatous plaques [41]. At the intracellular level, the first metabolic checkpoint is the Golgi apparatus, requiring microtubule indemnity [42]. Nevertheless, lysosomal degradation of HDL appears to be low [43]; thus, endocytic activity seems to be oriented towards selective lipid modification of HDL, lowering its CE and FC contents [40].

## 3. Serum HDL-C Levels and Cardiovascular Disease

Abundant clinical and epidemiological studies have consistently proved an inverse relationship between HDL-C concentration and cardiovascular risk (CVR) (Table 1). In the* Framingham Heart Study*, Wilson et al. described low HDL-C levels to entail a 3-4 times greater risk for cardiovascular-cause mortality, after following up over 2000 subjects aged >50 years for 12 years [12]. Moreover, the* Emerging Risk Factors Collaboration* workgroup have conducted a meta-analysis on 68 prospective studies, which supports this link between HDL-C and CVR following adjustment for other variables such as non-HDL cholesterol, TAG, and smoking [44]. Indeed, a 15 mg/dL increase in HDL-C levels appears to grant a 22% decrease in CVR, independently of TAG levels [44]. Similarly, the* Münster Heart Study* has reported dyslipidemias to be the factor most tightly correlated with coronary events, and HDL-C concentration in particular appears to be an important predictor of atherosclerotic disease [45, 46], in harmony with findings from the* INTERHEART Study*, which has also reported dyslipidemias—especially low HDL-C—to be key predictors of CVR, based on estimations with ApoB/ApoAI index [47].

Nevertheless, various clinical and genetic studies have suggested HDL-C not to be the best predictors of CVR in certain contexts (Table 2), as originally ascertained in reports on the effects of torcetrapib, a CETP inhibitor. Pilot studies offered very promising outcomes, describing 60–100% increases in HDL-C concentration with this drug [48]. These were later supported by a large multicentric clinical trial, the* ILLUMINATE* study, which found a 72.1% rise in HDL-C levels in subjects on this agent (*p* < 0.001) [16]. However, such increase was subsequently proved ineffective at deterring progression of atherosclerosis and associated with higher CVR (HR: 1.58; IC: 1.14–2.19; *p* = 0.006) [16, 49–51]. The trial suffered unforeseen premature termination due to a marked increase in subject mortality, despite the significant rise in HDL-C concentration. Although the causes behind this failure remain uncertain, the most prominent hypothesis attributes defects to torcetrapib and not CETP inhibition itself, presumably through direct vascular toxicity, promotion of hypertension, or interactions with the renin-angiotensin-aldosterone system [51]. Current studies aim at discerning the effects of other CETP inhibitors [52].

In the field of genetic research, studies in monozygotic twins have found 40–60% of variation in HDL-C levels to be genetically determined [53]. Roughly, 10 genes have been confirmed to participate in the regulation of HDL-C concentration in humans, almost all being responsible for Mendelian hereditary disorders that feature disturbances of HDL-C levels [54]. Research has also been directed at establishing associations between specific single-nucleotide polymorphisms (SNP) or haplotypes with either higher or lower HDL-C, in the presence or absence of atherosclerosis [55]. Variations of Apo A-I are among the most prominent: numerous mutations of the* APOAI* gene have been observed to propitiate a dose-dependent reduction in HDL-C levels, which may be virtually absent in homozygous individuals and 50% lower in their heterozygous counterparts [56]. Notoriously, although* APOAI* mutations lower HDL-C concentration, this is not consistently accompanied by higher CVR [57]. The R173C-Milano mutation represents an illustrative case in this respect: despite being linked to dramatically lower HDL-C levels, it appears to enhance their antiatherogenic assets, hinting at a gain-of-function effect [58].

Multiple other studies pinpoint the* ABCA-1* gene as the principal determinant of serum HDL-C levels [59]. Broad arrays of SNP have been identified for this gene, yet these rarely suppress its functionality [60]. Likewise, polymorphisms of the promoting gene of hepatic lipase have been found to impair its activity, thus significantly elevating HDL-C levels. Paradoxically, this genotype implicates a 40–50% increase in CVR [61]. Similarly, a SNP in the endothelial lipase gene (*LIPG 396Ser* allele) has also been shown to be linked with increased HDL-C concentration, without effect on CVR [62]. Finally, a mutation of Apo A-I has been described to be able to predict CVR even in absence of low HDL-C in the* Copenhagen City Heart Study* [63]. Therefore, Apo A-I may be a better predictor than the macromolecular complex in whole. Furthermore, because adequate HDL functionality strongly depends on Apo A-I, partial or total mutations of this component may considerably affect RCT [64] and, by extension, CVR. In the* Dallas Heart Study*, high cholesterol efflux capacity was inversely associated with cardiovascular mortality after adjusting for traditional cardiovascular risk factors, HDL-C concentration, and HDL particle concentration (HR = 0.33; 95% CI = 0.19–0.55; *p* < 0.05), whereas baseline HDL-C levels did not show such association in a similar model [65].

## 4. A Molecular Look into Dysfunctional High-Density Lipoproteins

The protein components of HDL constitute most of its complex macromolecular framework (55–60% of the particles' mass), accounting for structural apolipoproteins, enzymes, and their corresponding cofactors. The remaining proportion is composed of lipids, chiefly amphipathic in character (phospholipids and FC) [66]. These macromolecules show ample heterogeneity in regard to chemical composition, metabolism, and biologic activity, as a result of the continuous exchange of both apoproteins and lipids from the core occurring both in peripheral tissues and in circulation [66, 67]. This dynamism is enabled by the “flexibility” displayed by *α*-helices in Apo A-I in response to the structural modifications suffered in each subfraction [68].

**Figure 2:**
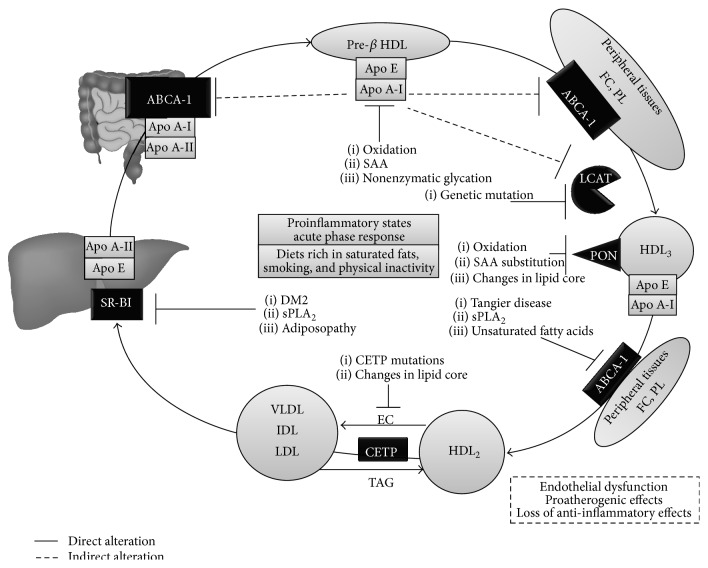
Key molecular checkpoints in HDL dysfunction. Genetic mutations, proinflammatory states, and the acute phase response are the main triggers for HDL dysfunction. For details, see the text. FC: free cholesterol; PL: phospholipids; ABCA-1: ATP-binding cassette transporter A-1; LCAT: Lecithin-Cholesterol Acyltransferase; PON: Paraoxonase; CETP: cholesteryl ester transfer protein; HDL: High-Density Lipoprotein; SR-B1: Scavenger Receptor B1; SAA: Serum Amyloid A; sPLA_2_: Secretory Phospholipase A_2_.

Furthermore, this heterogeneity in HDL structure is intrinsically related to their diverse functionality, where the specific protein contents—or proteome—of each subfraction are determinant [69]. This enables each subfraction to perform a particular activity, originating subgroups of particles with distinct cardiovascular effects [69]. Thus, alterations in key protein components yield molecules with abnormal functions or attenuated activity, the “dysfunctional HDL” [70]. These disruptions may stem from genetic or chronic proinflammatory environmental cues at various points in HDL metabolism (Figure 2). Indeed, although the acute phase response entails changes in lipid metabolism—aimed at evading injurious stimuli, for example, the lipopolysaccharide of Gram-negative bacteria—with certain atherogenic implications [71, 72], these effects are insignificant if the inflammatory stimulus is not prolonged [73, 74]. In contrast, disorders such as Coronary Artery Disease (CAD) and Type 2 Diabetes Mellitus (DM2) impose an intrinsic chronic inflammatory microenvironment at the endothelial level, triggering protein remodeling of HDL with the subsequent disruption of their antiatherogenic, antioxidant, and anti-inflammatory activities [74]. The molecular mechanisms involved in these phenomena are detailed in the following sections.

### 4.1. Protein Targets for HDL Dysfunction

**Figure 3:**
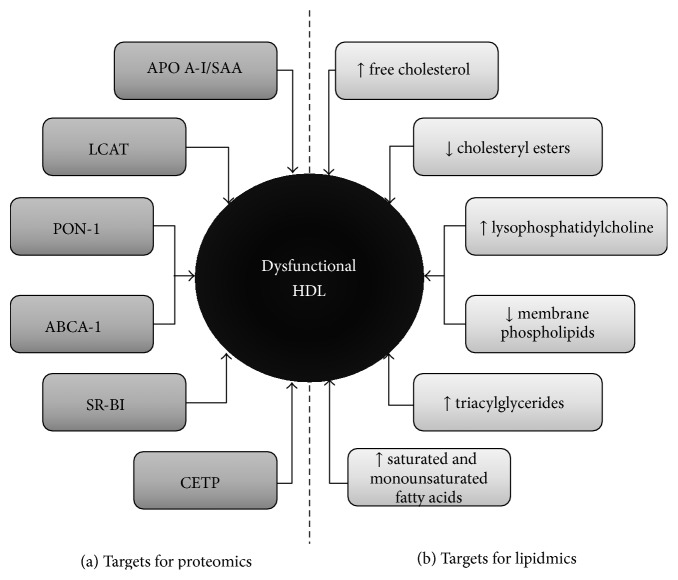
Targets susceptible to modification or alteration in HDL. The heterogeneity in HDL components renders these molecules very susceptible to alteration in various aspects. (a) depicts the protein targets, whereas (b) summarizes the lipid targets. These are studied by proteomics and lipidomics, respectively. HDL: High-Density Lipoprotein; SAA: Serum Amyloid A; LCAT: Lecithin-Cholesterol Acyltransferase; PON-1: Paraoxonase; ABCA-1: ATP-binding cassette transporter A-1; SR-B1: Scavenger Receptor B1; CETP: cholesteryl ester Transfer Protein.

Protein structures comprise the majority of components sensitive to modification in HDL (Figure 3(a)). Among these, Apo A-I—an apolipoprotein pivotal for stabilization of nascent HDL, as well as RCT by interacting with LCAT [20]—has amassed the greatest body of evidence [19]. This protein's functionality is disrupted in diseases such as DM2, where lysine residues are targets of nonenzymatic glycation, leading to generation of advanced glycation end-products [75]. This disturbance in protein structure hinders cholesterol efflux from various cells, including macrophages, towards HDL [75], inhibits their ability to interact with LCAT [76], and diminishes their anti-inflammatory activity in smooth muscle [77].

In a proinflammatory environment, Apo A-I becomes a substrate for myeloperoxidase (MPO), a hemoprotein released by macrophages and neutrophils that utilizes hydrogen peroxide (H_2_O_2_) and nitric oxide (NO) to catalyze oxidative reactions which yield nitrated reactive species. In turn, these mediators promote oxidative damage, especially on low-density lipoproteins (LDL) [78, 79]. These events have been demonstrated* in vivo*, with the detection of MPO in the atheromatous vascular lesions underlying systemic inflammatory states [80]. Likewise, oxidative dysfunction of Apo A-I interferes with LCAT activation, which is key for RCT and esterification of cholesterol contained in mature HDL [81]. MPO is involved in this mechanism as well: in the presence of elevated concentrations of hypochlorous acid (HOCl) or hydrogen peroxide, HDL_3_ become unable to activate LCAT [20]. This particular alteration involves amino acid residues 143–165 of Apo A-I, and particularly Met148, which is most sensitive to oxidizing, becoming methionine sulfoxide (Met[O]) [20, 82, 83]. Oxidation of these residues results in impaired reduction of CE hydroperoxides and PC hydroperoxides, which in physiological conditions would enhance the capture of HDL by hepatocytes [84, 85].

ABCA-1 interacts with Apo A-I by mediating the unidirectional efflux of cholesterol from foamy cells to HDL, preventing excess lipid accumulation in arterial walls [86]. These functions require interaction with completely functional Apo A-I [87]. Various reports describe MPO to act in the subendothelial space by oxidizing Tyr192, Tyr29, Tyr166, and Tyr236 (3-chlorotyrosine and 3-nitrotyrosine) and methionine residues of Apo A-I, disrupting its ability to transport cholesterol with ABCA-1 [88, 89]. Binding of Apo A-I to ABCA-1 and activation of Janus kinase 2 signaling are disrupted by these modifications [90], yielding proatherogenic HDL.

The ABCA-1/Apo A-I complex interacts with enterocytes for cholesterol absorption, HDL lipidation, and its subsequent release to lymphatic vessels [91]. In parallel to these events, HDL complexes also absorb vitamin E, lutein, and zeaxanthin, which are LDL-protecting antioxidant molecules that contribute to formation of nascent HDL [92, 93]. ABCA-1/Apo A-I activity is also fundamental for formation of nascent HDL (pre-*β*1 fractions) [94], representing a crucial point in the determination of their structure, as marks for further hepatic catabolism, and greater efficiency in RCT [95, 96]. Inflammation can disrupt all of these interactions, as oxidation of Apo A-I by MPO nullifies its function [97]. In addition, nitration and chlorination of HDL by MPO have been shown to prevent HDL from intervening in endothelial repair [98]. Furthermore,* in vitro* oxidation of HDL has been observed to promote activation of NF-*κ*B and expression of vascular adhesion molecules [99] and prevent this molecule from counteracting the vasoactive effects of oxidized LDL [100].

These enzymes are also susceptible to genetic alterations. Heterozygous subjects with defects in LCAT exhibit 36% lower HDL-C serum concentration, with higher levels of C-Reactive Protein, and greater intima thickness in the internal carotid arteries [101]. Similarly, in familial LCAT deficiency—an autosomal recessive hereditary disorder [102]—research has highlighted the presence of atherosclerosis in association with early death [103], along with a loss of the anti-inflammatory and antioxidant capabilities of HDL [104]. Certain genetic mutations have been described to disrupt ABCA-1, such as in Tangier syndrome, an autosomal recessive disease featuring a marked HDL deficit, lipid accumulation in macrophages, and accelerated atherosclerosis [105, 106]. Genetic alterations of SR-B1—which drives cholesterol from foamy cells in vascular walls to HDL and captures these particles in the liver [107]—also result in significant disturbances in lipid metabolism. SR-BI knockout mice have been found to suffer a greater risk for atherogenesis in spite of elevated HDL-C levels [108], as this yields decreased selective capture of CE in the liver [109, 110]. This receptor may also intervene in the effects of HDL on endothelial cells, by facilitating NO synthesis, hence promoting endothelial integrity [111]. In addition, dysfunctional HDL appear to contribute to endothelial damage in the setting of DM2. These altered HDL have been observed to diminish SR-BI expression and activity of its Akt-dependent signaling cascades [112], along with anomalous endothelial NO synthase activity [113], leading to endothelial dysfunction.

Both ABCA-1 and SR-BI are modulated by Secretory Phospholipase A_2_ (sPLA_2_), whose expression is augmented in chronic inflammation [114]. sPLA_2_ participates in various host defense and inflammatory mechanisms [115], belonging to a superfamily of enzymes able to hydrolyze glycerophospholipids at the sn-2 position, producing unsaturated fatty acids such as arachidonic acid, the major substrate for the synthesis of a myriad of messengers, including prostaglandins and leukotrienes [116]. sPLA_2_ is a paramount mediator within atheromatous plaque, triggering generation of multiple inflammatory intermediaries, oxidizing LDL, and promoting formation of foamy cells [117]. Research in transgenic mice has also shown sPLA_2_ to lower not only HDL-C serum levels but also its size and proportion of structural phospholipids [118]. Likewise, sPLA_2_-mediated modification of HDL components has been observed to disrupt the cholesterol efflux associated with decreased expression of ABCA-1, independently of Serum Amyloid A (SAA) concentration [119], possibly through inactivation of LXR transcription factor.

SAA is an acute phase protein synthesized in the liver, which acts in close association with the HDL_3_ subpopulation [120]. During the acute phase response, circulating SAA displaces Apo A-I and incorporates into the lipoprotein membrane, becoming the major protein component of HDL (~80%) [121]. This leads to lower HDL-C concentration by impeding ABCA-1-mediated lipidation of Apo A-I, with reduced formation of nascent HDL [122], along with increased free circulating Apo A-I and TAG, and decreased Paraoxonase-1 (PON-1) levels [123]. These dysfunctional, SAA-rich lipoproteins present a proteoglycan-binding domain—which facilitates its retention in arterial walls—and have lower cholesterol efflux capacity. Indeed, proteomic analyses have demonstrated that HDL's cholesterol efflux capacity is inversely correlated with HDL SAA1 and SAA2 [124].

PON-1—an HDL-bound arylesterase, able to hydrolyze several oxidized or altered lipids, protecting HDL from lipid peroxidation [125]—is another target of modification. Decreased PON-1 activity has been proposed to yield dysfunctional HDL, favoring premature atherosclerosis [126], ostensibly through oxidative stress [127] and production of advanced glycation end-products in hyperglycemic milieus, as seen in DM2 [128], as well as in obese and hyperlipidemic individuals, also characterized by significant inflammation and oxidative stress [129, 130]. Furthermore, MPO, PON-1, Apo A-I, and HDL can form a ternary complex which further potentiates inflammation, where MPO oxidizes tyrosine residue 71 in PON-1, inhibiting its antioxidant function [131].

Lastly, activity of CETP—responsible for exchange of CE from HDL to TAG-rich lipoproteins (i.e., VLDL, LDL, and IDL), resulting in TAG-rich HDL [132]—can also be downregulated, with beneficial effects, as seen in subjects with the TaqIB polymorphism in the* CETP* gene, who boast a significantly lower CVR [133]. However, different variants of this gene result in radically distinct phenotypes. In a Japanese population, a G-to-A mutation at the 5′ splice donor site of intron 14 in the* CETP* gene has been identified to result in hyperalphalipoproteinemia associated with increased CVR [134, 135]. Although the mechanisms underlying this divergence remain incompletely elucidated, it appears these TAG-rich HDL are unable to promote an adequate efflux of cholesterol from foamy cells [136].

### 4.2. Lipid Targets for HDL Dysfunction

Although the main targets for modification linked with HDL dysfunction are protein in nature, these may also be affected by changes within its lipid core (Figure 3(b)). Quantitatively, phospholipids (chiefly PC and sphingomyelin) are the main constituents of the HDL lipidome (40–60%), followed by CE (30–40%), TAG (5–12%), and FC (5–10%) [137]. These components are spatially organized according to their biochemical properties: phospholipids and FC form an outer hydrophilic monolayer which encloses a hydrophobic core rich in CE and TAG [138]. HDL also carry other lipids with important qualities, including sphingosine-1-phosphate—which is antioxidant and regulated vascular tone and endothelial function [139]—and liposoluble vitamins [140].

Dysfunctional HDL exhibit 25% less lipids per milligrams of protein, reflecting lower contents of sphingomyelin, phosphatidylinositol, and PC, higher concentration of lysophosphatidylcholine (LPC) and FC, and a substitution of 50% of CE for TAG [141]. These lipid changes can alter antiatherogenic HDL assets. The reorganization of lipid components caused by upregulated CETP activity—as seen in states of insulin resistance—alters the CE/TAG ratio in HDL, which is fundamental for their antioxidant activity and circulation [142]. Higher TAG contents in the lipid core also impair transfer of CE through SR-BI, hindering RCT [143].

In addition, during acute inflammation, PLA_2_ subtype IIA is activated, hydrolyzing HDL phospholipids and culminating in accumulation of deleterious oxidized fatty acids [144], which disrupt the secondary and tertiary structure of Apo A-I [145]. In addition, hydrolysis of phospholipids in the superficial monolayer of HDL—for example, by sPLA_2_ [146, 147]—leads to redistribution of CE from the core towards the surface monolayer, reducing its fluidity, impairing cholesterol efflux in RCT [148], potentiating release of FC to peripheral tissues [149], and diminishing antioxidant activity [150].

The loss of this protection is particularly important in atherosclerosis, where HDL may prevent LDL oxidation [151], a critical step in the progression of the atheroma, as oxidized LDL is a powerful inducer of monocyte recruitment to the subendothelial space [152]. LDL oxidation also generates biologically active phospholipids derived from arachidonic acid, which intervene in chemotaxis and monocyte migration [153], through expression of MCP-1, M-CSF, and IL-18 [154]. In addition, sPLA_2_ released by activated macrophages modifies the lipid structure of HDL, hydrolyzing phospholipids in their external monolayer [155], yielding a great quantity of unsaturated fatty acids and modified phospholipids, such as LPC [156], a proinflammatory component of atheromatous plaques [157]. LPC has been found to play an active role in atherosclerosis, acting on various cells—monocytes, macrophages, and endothelial and smooth muscle cells—generating oxidative stress, and promoting chemotaxis through expression of adhesion molecules and inflammatory messengers, including IL-1*β*, INF-*γ*, and MCP-1 [158]. In subjects with CVD, higher levels of these proinflammatory lipids in HDL have been related to lower cholesterol efflux capacity [159] and attenuated PON-1 activity, inhibiting the antioxidant ability of HDL and turning HDL into proinflammatory agents [160]. Similarly, in DM2, these oxidized lipids generate dysfunctional HDL with altered anti-inflammatory and antioxidant activity [161].

Higher levels of unsaturated fatty acids in HDL can also deteriorate ABCA-1 functionality, inhibiting its expression in cell membranes and augmenting its degradation ratio, with the consequent reduction in cholesterol efflux from vascular spaces to HDL, thus favoring atherogenesis [162]. In addition, unsaturated fatty acids may repress LXR/RXR transcription factors—which promote ABCA-1 synthesis [163]—and activate Protein Kinase C*δ*, which phosphorylates serine residues in ABCA-1, destabilizing its structure [164].

### 4.3. Implications of Environmental Factors on Dysfunctional HDL

Despite the ample diversity of genetic alterations that can lead to HDL deficiency or dysfunction [165], environmental elements also play an important role in these phenomena, as well as various conditions and diseases. Notably, in systemic lupus erythematosus and rheumatoid arthritis—both autoimmune diseases linked to accelerated atherosclerosis—dysfunctional HDL have been observed to be unable to prevent LDL oxidation* in vivo* [166, 167]. Likewise, in DM2, persistent hyperglycemia leads to structural changes through glycation of Apo A-I, and other alterations caused by chronic inflammation, including a quantitative reduction in Apo A-I, and increased SAA density [168].

Nascent HDL must undergo a process denominated “lipidation” in order to maintain a proper structure for RCT, which happens mainly in hepatocytes and enterocytes [169]. Nevertheless, adipocytes may also modulate HDL function by transferring cholesterol to these lipoproteins [170], possibly mediated by ABCA-1 and SR-BI in these cells [171]. Indeed, Zhang et al. [170] have demonstrated this premise both* in vivo* and* in vitro*, describing mature adipocytes to transfer cholesterol to HDL in a fashion similar to that of macrophages. Disorders such as obesity, insulin resistance, and DM2 feature profound disruptions of adipocyte physiology [172], propitiated by proinflammatory circumstances with high TNF-*α* levels, which appear to inhibit cholesterol efflux to these cells from HDL, representing a possible contributing factor to low HDL levels in these disorders.

Nutritional factors also influence HDL functionality. In a study by Nicholls et al. [173], subjects on saturated fat-rich diet showed attenuated anti-inflammatory HDL activity 6 hours after intake, whereas individuals on a diet rich in unsaturated fats displayed opposite effects, despite both diets being isocaloric. Likewise, long-term resistance training has been associated with improved redox activity of HDL in young subjects, independently of body weight [174]. In contrast, smoking triggers HDL dysfunction, by hindering functionality of LCAT, CETP, and hepatic lipase, as well as promoting oxidative stress [175]. Therefore, future research should focus on further discerning the impact of these and other environmental factors on HDL, in order to establish pertinent recommendations for the opportune management and prevention of the metabolic disturbances they may promote.

## 5. Proteomics and Lipidomics: A Focus on HDL

HDL participates in an extensive catalogue of intricate pathophysiologic cascades, which demand specialized approaches for their molecular study. Among these stands proteomic research, which employs diverse molecular techniques for detailed description of the structure and function of proteins, in order to discover novel biomarkers and/or therapeutic targets in the diagnosis and treatment of human pathologies and increase our understanding of the underlying biologic processes [176]. On the other hand, lipidomics aim to quantitatively define lipid classes in various biologic systems and characterize their cellular distribution through procedures like mass spectrometry, permitting clarification of the cellular processes in which they intervene [177]. Its application is noteworthy in conditions such as DM2, neurodegenerative disorders, and cystic fibrosis.

In the setting of HDL metabolism, both tools have been utilized for evaluation of functionality, amplifying knowledge of their role in RCT and anti-inflammatory and antioxidant activity. Prominent aspects include protection against LDL oxidation [129], endothelial homeostasis [111], repression of vascular adhesion molecules [178], and inhibition of platelet aggregation [179]. Proteomics and lipidomics may be particularly useful at identifying the molecules associated with HDL that intervene in their inverse relationship with CVR, as quantitative assessment of HDL-C fails to fully explain this premise [180]. These methods may drive a shift in the classical categorization of HDL subfractions, from the established physicochemical classification towards a new model based on their physiologic activity and pathophysiologic roles [181–183].

Many techniques are currently utilized; among these, mass spectrometric immunoassay is a high-performance protein analysis method that unites immunoaffinity with the power of mass spectrometry, in order to identify the components of chemical structures [184]. This allows protein isolation and quantification of molecular variants, including changes in primary structure and posttranslational modifications [185]. However, scarce availability of highly purified specific antibodies, elevated costs, problems with immunoaffinity in human samples, and the presence of autoantibodies have disfavored this technique [186, 187], paving the way for other methods, such as Selected Reaction Monitoring and Parallel Reaction Monitoring, which allow simultaneous isolation of multiple proteins in complex samples [186]. The former, also called Multiple Reaction Monitoring, is known for its good performance in quantification of various proteins in highly complex and heterogeneous samples, allowing realization of the analysis in a single programmed step [188]. These methods are very efficient for protein quantification, with similar linearity, dynamic range, precision, and repeatability [189]. Nonetheless, Parallel Reaction Monitoring currently appears to be the best method, by virtue of its relative simplicity and greater specificity—due to its use of isotope marking [189]—and its reproducibility is apt for great-scale widespread application [190].

These methods suffer certain limitations. For example, they require knowledge of the molecular weight of the peptide analyte and its fragmentation pattern; thus, sensitivity may be diminished in samples with high protein content without a preliminary analysis [191]. In this case, complementary procedures that reduce this abundance of protein may aid in preserving sensitivity. These include multiplexed immunoassay panels (Multianalyte Profiling), which complements Multiple Reaction Monitoring, assuring adequate sensitivity for quantitative protein analysis [191].

### 5.1. Contributions of Proteomics and Lipidomics to HDL Research

Although initial studies exploiting these techniques in this setting were centered on proteins already known to be associated with HDL and lipid metabolism [192], further research managed to identify several other proteins previously unknown to be related to HDL, including complement factors and other immune and coagulation intermediaries [193]. Likewise, differing protein expression patterns were described for HDL_2_ and HDL_3_, raising interest in the functional roles for each subfraction [192].

These tools have allowed identification of a wide array of proteins involved in inflammation, such as components of the complement system (C3, C4, and C5), vitronectin, clusterin (Apo J), and HDL-associated endopeptidases [66]. The complement system has been observed to directly participate in inflammation in atheromas [194], regulated by vitronectin and clusterin [195]. Likewise,* in vitro* assays have shown HDL can prevent organization of the membrane attack complex [196, 197] and share a negative correlation with C5b-9 levels [198], promoting complement inhibition.

HDL also appears to act as platforms for organization and mobilization of immune responses. Extracellular vesicles in close association with these lipoproteins have been described to contain a diversity of immunity-related microRNA molecules and complement-activating proteins [199–201]. More selectively, HDLs are also anchors for the organization the trypanosome lytic factor, a macromolecular complex containing Apo L1 and haptoglobin-related protein, highly lytic for* Trypanosoma brucei* [202]. This complex binds to HDL through an 18-amino acid signal peptide in its N-terminal region, interacting with lipids in the lipoprotein's monolayer [203]. This complex mediates hemoglobin binding and endocytosis of the parasite, facilitating its lysis and impeding progression of this infection [204].

Furthermore, HDLs have been proved to induce a shift in macrophage phenotype, decreasing expression of proinflammatory mediators such as INF-*γ*, iNOS, IL-6, and TNF-*α* and potentiating the expression of IL-4 and markers typical of anti-inflammatory M2 macrophages, including Arg-1, Receptor, CD163, Fizz-1, and YM-1, through JAK/STAT6-dependant cascades [205]. In contrast, individuals with subclinical atherosclerosis exhibit dysfunctional HDL with lower PON-3 activity, reducing their antioxidant capacity in the atherosclerotic plaque [206]. Furthermore, HDLs of individuals with DM2 or established CVD carry proteins such as ENRAGE, MPO, and plasminogen activator inhibitor-1, promoting atherosclerosis [191].

Finally, the precision of proteomics and lipidomics has allowed characterization of the local distribution of various kinds of molecules within the atherosclerotic plaque [207, 208], allowing further molecular differentiation between stable and unstable plaques, extending our comprehension on the processes leading to plaque rupture [209, 210]. Stable plaques display higher levels of superoxide dismutase 2 and fibrinogen fragment D—with antioxidant effects [211]—and participate in smooth muscle contraction [212], respectively, and lower expression of glutathione S-transferase, Rho GDP-dissociation inhibitor 1, HSP20, and HSP27, which defend against electrophile reactants in vessel walls [213], regulate smooth muscle tone [214], and modulate cell growth and motility [215], respectively. This evidence posits a role for HDL in innate immunity and novel mechanisms in chronic inflammation and atherosclerosis [208, 216].

### 5.2. Proteomics, Lipidomics, Cardiovascular Risk, and Clinical Applications

Decades ago, HDL_2_ and HDL_3_ subfractions were first obtained through ultracentrifugation, representing the gold standard method for this objective [217]. These subfractions have been studied regarding their ability to predict CVR and whether assessment of their biologic behavior is comparable to evaluation of total HDL-C concentration. In multiple studies, HDL_3_ have been inversely related to CVR—more strongly than HDL_2_ [218, 219]—even in patients with established CVD [220]. Because these subfractions are susceptible to isolation and quantification based on their physicochemical properties, proteomics may propel the proposal of new, more sensitive CVR markers, as well as facilitating assessment of their reproducibility in the clinical setting [221] (Table 3).

These techniques have revealed qualitative alterations in HDL in subjects with CVD [66, 222–224], DM2 [225], chronic kidney disease [226–228], and rheumatoid arthritis [229] in comparison to healthy populations, often without significantly lower HDL-C concentrations [66, 222]. Gene ontology has allowed exploration of these changes. In these scenarios, proinflammatory HDL-associated proteins are upregulated—for example, SAA2, C5, histone H1, and fibrinogen *β*-chain binding to HDL—whereas lipid metabolism-related HDL-associated proteins are downregulated, including Apo C-I, Apo C-II, Apo E, LCAT, and PLTP [223, 230]. Apo A-I is also vulnerable to numerous modifications; in particular, oxidation of residue M148 has been extensively documented in CVD and DM2 [231]. Expression of Apo E in HDL_3_ also appears to be increased in individuals with CVD [66], and HDL-bound Apo C-III has been related to higher CVR [232]. Nonetheless, SAA may yield greater clinical utility. This protein has been described to be better predictor of clinical outcomes in non-ST-segment elevation acute coronary syndrome in comparison to C-Reactive Protein [233]; and the quantity of HDL-bound SAA has been correlated with levels of inflammatory markers [230].

Many other proteins may bind to HDL, most likely by hepatic remodeling or interactions in peripheral tissues, with significant impact in atherogenesis [234, 235]. Future studies should aim to more thoroughly characterize the role of these HDL-associated proteins in the functionality of this lipoprotein, their clinical utility, and their potential as therapeutic targets. Promising candidates for this research include the fatty acid-binding protein, hemoglobin, HLA-A43 [223], rab7b, *α*-1-antitrypsin, Serum Amyloid P component [222], *α*1-acid glycoprotein 1, zinc-*α*2-glycoprotein, surfactant-associated protein B (SP-B), c-src, complement factor D [230], complement factor B, complement components 4B and C1s, vitronectin, and prothrombin [234].

Proteomics also allows continuous observation of structural and functional changes in HDL in response to pharmacological intervention, as executed by Green et al. [236], who found combined statin/niacin therapy to improve HDL_3_ composition in patients with established CVD, with a decrease in Apo E contents (*p* = 0.02) and an increase in PLTP concentration (*p* = 0.02) after a year on treatment, thus representing a structural shift in this subfraction towards an architecture resembling that found in healthy subjects. Another interesting aspect in this field is the prediction of complications during treatment. The expression of arachidonate 5-lipoxygenase-activating protein—whose increased activity in muscle is a hallmark of simvastatin use—may be monitored through proteomics, allowing titration of doses in order to avoid statin-associated toxicity [237]. Likewise, proteomics may help decipher the causes of failure of certain therapeutic trials, as seen in the* Fibrate Intervention and Event Lowering in Diabetes* (FIELD) substudy [238]. In this report, no benefit was ascertained for 200 mg fenofibrate OD versus placebo regarding CVR [238]. Ulterior analysis showed subjects in this regimen to have higher Apo A-II levels and lower PON and PTLP activity in HDL, as well as compositional changes in lipid conglomerates, such as increased LPC and decreased sphingomyelin, favoring LCAT disruption [239] and slowing maturation of HDL [240]. This kind of treatment evaluation may also be useful in surgical scenarios, as in the case of subjects who have undergone closure of patent foramen ovale, where an increase in cholesterol efflux and a decrease in lipid oxidation can be detected after surgery, with cardiovascular benefits [241]. Similarly, patients with >70% stenosis of carotid arteries have been shown to have higher SAA proportions in HDL when compared to normolipemic subjects (*p* = 0.003), confirmed by western blot analyses [224].

Lastly, molecular HDL analysis not only facilitates therapeutic response analysis but may also assist in application of therapeutic measures involving ligand-receptor interactions. Apo A1 expression has been experimentally upregulated in HDL by exploiting an HDL-conjugated chimeric IL-15 fusion protein and Sushi domains in this interleukin, resulting in stimulation of T lymphocytes and NK cells [242]. Similarly, anchoring Apo A1 to IFN-*α* has been reported to potentiate cytotoxic T lymphocytes and attenuate side effects of IFN-*α* monotherapy [243]. These innovative findings profile proteomics as an important tool in the study of HDL as vehicles for immunomodulation.

On the other hand, research in lipidomics has found significant associations between HDL lipid composition and cardiometabolic disease. Lower phospholipid proportions in dysfunctional HDL have been reported to impair cholesterol efflux [226], even independently of SAA concentration [114]. Lipidomics has also found differences in HDL constitution depending on severity of CVD. Concentrations of PC, unsaturated fatty acids, omega-3 fatty acids, and sphingomyelin appear to be significantly higher in earlier stages of CVD than in more severe cases [244]. Other changes in lipid composition of HDL have been found in CVD and DM2, including higher TAG, saturated fatty acids, diallyl fatty acids, linoleic acid [245], LPC, palmitate-rich triacylglycerols, and diacylglycerols [225], as well as increased products of lipid peroxidation such as 5-HETE, 12-HETE, 15-HETE, 13-HODE, and 9-HODE [246].

## 6. Conclusions

HDL is one of the most biologically variable molecules, with a great heterogeneity dictated by specific groupings of proteins or lipids, suggesting the existence of various subfractions with distinct functional profiles. Although their main role involves prevention of cholesterol accumulation in peripheral tissues, their participation in beneficial processes is hardly limited to this aspect. Multiple pathologic conditions trigger structural and functional alterations in HDL, becoming proinflammatory molecules unable to maintain endothelial homeostasis, thus becoming “dysfunctional HDL.” To elucidate this dysfunctionality, detailed research at a molecular level is required on the various components associated with these lipoproteins, uncovering new therapeutic alternatives regarding progression of atherosclerosis. To this end, proteomics and lipidomics appear to be the most promising methods in the exploration of the physiologic, pathologic, and potentially therapeutic roles of HDL.

## Figures and Tables

**Table 1:** HDL-C as a good predictor of cardiovascular risk.

Author [reference]	Methodology	Conclusions
Barter et al. [6]	Post hoc analysis of data from the *Treating to New Targets *Study, a multicentric, randomized, double-blind study which assessed the predictive value of HDL-C in 9,770 subjects with established CVD on statin therapy.	Based on HDL-C quintiles, a multivariate analysis revealed individuals with HDL-C >55 mg/dL to have a lower risk of cardiovascular mortality than subjects with HDL-C <38 mg/dL (HR: 0.75; IC 95%: 0.60–0.95). In subjects on statin therapy, the best lipid predictor for CVD was HDL-C, even when LDL-C <70 mg/dL.

Castelli et al. [10]	Multicentric case-control study with 6859 subjects of diverse ethnicities from the *Cooperative Lipoprotein Phenotyping *Study.	HDL-C concentration was significantly higher in subjects without established CVD. An inverse correlation was ascertained between these factors, without significant variation after adjustment for total cholesterol, LDL-C, and TAG levels.

Gordon et al. [11]	Prospective report from the *Framingham Heart *Study, with 2,815 subjects aged 49–82 years, whose serum lipids were quantified throughout a 4-year follow-up period in order to evaluate cardiovascular mortality.	142 individuals developed CVD (79 males, 63 females), with HDL-C being the best CVR predictor. These variables shared an inverse correlation in both genders, even after adjustment for multiple other risk factors.

Wilson et al. [12]	Prospective report from the *Framingham Heart *Study, with 2,748 individuals aged 50–79 years after a 12-year follow-up period.	An inverse relationship was identified between HDL-C levels and coronary artery disease mortality in both genders (*p* > 0.05). HDL-C was found to be the best predictor of cardiovascular mortality.

Emerging Risk Factors Collaboration [44]	302,430 subjects from the *Emerging Risk Factors Collaboration* Study data without history of coronary artery disease were studied to analyze the association between serum lipids and CVR.	A strong inverse association was found between risk of coronary artery disease and HDL-C levels after adjusting for nonlipid risk factors (HR: 0.71; IC 95%: 0.68–0.75) and even after adjustment for non-HDL cholesterol (HR: 0.78; IC 95%: 0.74–0.82).

Assmann et al. [45]	The incidence of coronary artery disease was determined in 4,559 male subjects aged ≥40 years from the *Prospective Cardiovascular Münster* Study over a 6-year follow-up period.	Univariate analysis revealed a significant inverse relationship between CAD and HDL-C (*p* > 0.001), even after adjustment for several other risk factors.

**Table 2:** HDL-C as a poor predictor of cardiovascular risk.

Author [reference]	Methodology	Conclusions
Barter et al. [16]	Randomized, double-blind study on 15,607 subjects with high CVR, who received (a) atorvastatin + torcetrapib or (b) atorvastatin + placebo.	Although treatment with torcetrapib raised HDL-C 72% from the baseline (*p* < 0.001), it entailed an increase in cardiovascular mortality in these subjects (HR: 1.25; 95% CI: 1.09–1.44; *p* = 0.001).

Nissen et al. [49]	Prospective, multicentric, randomized, double-blind study on 1,188 patients with CAD who underwent intravascular ultrasonography and received (a) atorvastatin + torcetrapib or (b) atorvastatin + placebo.	Subjects on atorvastatin + torcetrapib had a 61% increase in HDL-C and a 20% decrease in LDL-C levels when compared to the group on atorvastatin + placebo. However, the former also suffered a greater rise in blood pressure (21.3% versus 8.2%) and incidence of hypertensive cardiovascular events (23.7% versus 10.6%), without significant differences in progression of atherosclerosis, as evaluated by intravascular ultrasonography.

Kastelein et al. [50]	850 heterozygotes with familial hypercholesterolemia were treated with 20, 40, or 80 mg of atorvastatin for a 4-week period, followed by (a) atorvastatin monotherapy or (b) atorvastatin + torcetrapib 60 mg for 24 months, and underwent ultrasonography for evaluation of intima-media thickness.	HDL-C levels were significantly higher in the atorvastatin + torcetrapib group (81.5 ± 22.6 mg/dL versus 52.4 ± 13.5 mg/dL; *p* < 0.001), who also displayed lower LDL-C and TAG concentrations. Nevertheless, those on monotherapy were found to have greater intima-media thickness in the common carotid artery.

Voight et al. [62]	Mendelian randomization study which evaluated the association between the *LIPG* Asn396Ser SNP and incident myocardial infarction in 50,763 participants from six prospective cohort studies and case-control studies involving an additional 16,685 cases of myocardial infarction and 48,872 controls and proposed a genetic score combining 14 common SNP that exclusively associate with HDL cholesterol and then tested this score in up to 12,482 cases of myocardial infarction and 41,331 controls.	The *LIPG* Asn396Ser allele had a prevalence of 2.6% and was associated with increased HDL-C, without effect on LDL-C y TAG. In meta-analysis, carrier status for Asn396Ser was associated with an increase of roughly 0.29 SD units in HDL-C (*p* = 8 × 10^−13^), with no associations to other risk factors. Nevertheless, this allele was not associated with myocardial infarction (OR: 0.99; 95% CI 0.88–1.11, *p* = 0.85), without significant heterogeneity among the studies included (*p* > 0.05). Finally, a 1 SD increase in HDL-C due to genetic score was not associated with risk of myocardial infarction (OR: 0.93; 95% CI: 0.68–1.26, *p* = 0.63).

Haase et al. [63]	The APOA1 gene was resequenced in 190 subjects, evaluating the effects of mutations on HDL-C levels, risk of ischemic heart disease, myocardial infarction, and mortality in 10,440 individuals from the prospective *Copenhagen City Heart *Study, who were followed for 31 years. Results were validated in an independent case-control study with 16,035 subjects.	The A164S mutation was found to be a predictor of ischemic heart disease (HR: 32; 95% CI: 1.6–6.5), myocardial infarction (HR: 5.5; CI 95% 2.6–11.7), and mortality (HR: 2.5; 95% CI: 1.3–4.8) in heterozygotes, in comparison to noncarriers. A164S heterozygotes also showed normal levels of Apo A-I, as well as HDL-C and other serum lipids.

Rohatgi et al. [65]	Multiethnic, population-based cohort study on 2,416 adults free from CVD who were participants in the *Dallas Heart *Study, where the association between cholesterol efflux capacity and CVD incidence was assessed.	HDL-C levels were found to be unrelated to CVD incidence after adjustment for traditional cardiovascular risk factors. Cholesterol efflux capacity was associated with lower CVR, even after adjustment for HDL-C concentration, HDL particle concentration, and traditional cardiovascular risk factors (HR: 0.33; 95% CI: 0.19–0.55).

Sirtori et al. [247]	21 subjects with the Apo A-IMilano mutation were compared with age- and sex-matched control subjects from the same kindred and with 2 series of matched subjects with primary hypoalphalipoproteinemia (HDL-C levels under the 10th percentile for their gender and age), regarding ultrasonographic findings in carotid arteries.	Subjects with hypoalphalipoproteinemia had greater intima-media thickness (0.86 ± 0.25 mm) than the control group (0.64 ± 0.12 mm) and subjects with the Apo A-IMilano mutation (0.63 ± 0.10 mm); *p* < 0.005. Moreover, subjects with hypoalphalipoproteinemia had a significantly higher prevalence of atherosclerotic plaques than both of the other groups, despite the lower HDL-C levels (19.8 ± 9.8 mg/dL, *p* < 0.05).

Schwartz et al. [248]	Randomized, single-blind study on 15,781 subjects with recent diagnoses of acute coronary syndrome who received (a) dalcetrapib 600 mg daily or (b) placebo.	Subjects on dalcetrapib had a 31–40% increase in HDL-C levels, with minimal effects on LDL-C. Compared to placebo, the dalcetrapib group did not show significantly higher CVR (HR: 1.04; 95% IC: 0.93–1.15, *p* = 0.52).

**Table 3:** Contributions of proteomics and lipidomics to cardiovascular risk estimation.

Author [reference]	Methodology	Results
Vaisar et al. [66]	7 males with established CVD were compared with 6 healthy, age-matched subjects, whose HDL-C, HDL_3_, and HDL-associated proteins were studied.	No significant differences in HDL-C concentration were found between groups (40 ± 11 mg/dL versus 45 ± 12 mg/dL, resp.). In individuals with CVD, the proteins most commonly found associated with HDL_3_ were Apo C-IV, PON-1, C3, Apo A-IV, and Apo E. HDL_3_ of control subjects were found to have increased levels of clusterin and vitronectin.

Tan et al. [222]	40 subjects with established CVD were compared to 40 healthy subjects, who had their HDL_3_ and HDL_2_ studied quantitatively and qualitatively.	No significant differences in HDL-C concentration were found between groups. However, in subjects with CVD, HDL_3_ were found to be rich in Apo E, Apo A-I, Apo A-IV, Apo L1, Serum Amyloid P component, PON-1, *α*-1B glycoprotein, and vitamin D-binding protein, along with low Rab levels. Likewise, HDL_2_ were found to have low levels of Apo A1, Apo E, PON-1, Apo L1, haptoglobin, serotransferrin, Rab7, and complement factor B, along with increased Serum Amyloid P component, *α*-1 antitrypsin, and acid ceramidase.

Yan et al. [223]	Case-control study comprising 10 males with chronic heart disease versus 10 healthy subjects matched by age, Body Mass Index, and lipid profiles, who had their HDL composition studied for comparison.	12 HDL-associated proteins differed significantly between subjects with chronic heart disease and healthy individuals, most of which participate in lipid metabolism. Gene ontology analysis revealed proteins involved in inflammation and other immune responses (SAA, C5, histone H1, and fibrinogen beta chain) to be differentially upregulated, whereas proteins involved in lipid metabolism (Apo C-I, Apo C-II, and fatty acid-binding protein) were differentially downregulated. Further ELISA analysis supported these findings, confirming higher SAA and lower Apo C-I in subjects with chronic heart disease versus healthy subjects (126.5 ± 67.3 *μ*g/mg versus 68.7 ± 12.4 *μ*g/mg, *p* = 0.024; and 68.8 ± 14.4 *μ*g/mg versus 81.1 ± 10.6 *μ*g/mg, *p* = 0.040, resp.).

Lepedda et al. [224]	The apolipoproteins of 79 patients undergoing carotid endarterectomy (due to stenosis >70%) were isolated and compared with those from 57 normolipemic subjects.	Apo A-I, Apo C-II, Apo C-III, Apo E, Apo D, and SAA were found to be associated with HDL. Only SAA was found to display a significant differential distribution, being more abundant in the group undergoing carotid endarterectomy (*p* = 0.045). SAA may be a CVR marker reflecting HDL quality.

Holzer et al. [226]	HDL was isolated from end-stage renal disease patients on maintenance hemodialysis (*n* = 27) and healthy subjects (*n* = 19); proteomic techniques allowed identification of HDL-associated proteins in both groups.	Patients on hemodialysis had lower levels of HDL-C (61 mg/dL versus 43 mg/dL, *p* < 0.01). 35 HDL-associated proteins were identified, most abundantly Apo A-I and Apo A-II. SAA was found only in the HDL of patients on hemodialysis. 9 proteins were found to be significantly altered in this group, including SAA. In addition, HDL of this group displayed lower proportions of phospholipids and higher proportions of LPC.

Mangé et al. [227]	A quantitative proteomic analysis was realized in 23 patients on hemodialysis and 23 age-matched control subjects.	Individuals on hemodialysis showed significantly lower HDL-C and serotransferrin levels, along with increased expression of Apo C-II and Apo C-III (with greater Apo C-II/Apo C-III ratio), which may act as markers of HDL maturity.

Weichhart et al. [230]	HDL was isolated from patients with end-stage renal disease and healthy subjects through sequential ultracentrifugation. Shotgun proteomics was used to identify HDL-associated proteins in a uremia-specific pattern.	Gene ontology functional analysis showed that in the group with end-stage renal disease, HDL-associated proteins involved in lipid metabolism were disrupted (including Apo A-I, Apo E, Apo A-IV, PON-1, LCAT, and PLTP). Instead, their HDL were found to be rich in surfactant protein B, Apo C-II, SAA, and *α*-1-microglobulin, representing a possible explanation for the increased inflammation and cardiovascular mortality seen in uremia.

Yassine et al. [231]	11 subjects with DM2, 15 with DM2 plus established CVD, and 8 control subjects had their HDL isolated in order to determine relative ratios of oxidation of the M148 residue of Apo A-I.	Patients with DM2 plus CVD displayed significantly lower levels of HDL-associated Apo A-I when compared to subjects with DM2 only (84 ± 39 versus 90 ± 40; *p* < 0.05). Molecular methods allowed determination of a relative oxidation ratio of the M148 residue in Apo A-I. This ratio was significantly higher in the groups with DM2 and CVD (0.236 ± 0.084) and DM2 only (0.127 ± 0.037), in comparison to the control group (0.087 ± 0.02); and *p* < 0.05.

Jensen et al. [232]	173,230 subjects from the *Nurses' Health Study (NHS)* and the *Health Professionals Follow-Up Study (HPFS)*, who had their levels of Apo C-III-associated and non-ApoC-III-associated HDL quantified and evaluated in regard to CVR.	HDL-C concentration was negatively correlated with CVR in both studies (IRR: 0.78; 95% IC: 0.63–0.96, *p* = 0.02). Nevertheless, increased levels of non-Apo C-III-associated HDL were negatively associated with CVR (IRR: 0.66; 95% IC: 0.53–0.83, *p* = 0.0001), whereas increased levels of Apo C-III-associated HDL were positively associated with CVR (IRR: 1.18; 95% IC: 1.03–1.34, *p* = 0.001).

Ståhlman et al. [225]	Mass spectrometry was used to characterize the lipidome of 3 groups of women from the *DIWA* study: (a) control group; (b) DM2 + insulin resistance + dyslipidemia; (c) DM2 + insulin resistance + normolipemia.	Smaller HDL particles were found in the dyslipidemic group, with increased LPC (13%) palmitate-rich triacylglycerols and diacylglycerols (77%) possibly reflecting enhanced CETP activity. The subjects also displayed a high Apo A-I/plasmalogen ratio compatible with oxidative stress seen in DM2.

Kostara et al. [244]	Case-control study with 60 subjects with normal coronary arteries and 99 patients with established CVD grouped by severity of coronary artery stenosis (mild, moderate, and severe). Lipidomic analysis assessed patterns in the constitution of HDL in each group.	HDL-C was significantly lower in the mild disease group versus severe disease group (43.6 ± 10.9 mg/dL versus 38.4 ± 6.8 mg/dL). Subjects with CVD had higher proportions of saturated fatty acids, phospholipids, triacylglycerides, and cholesteryl esters in HDL in comparison to controls, along with lower proportions of sphingomyelin and phosphatidylcholine. Likewise, subjects with mild disease had greater proportions of phosphatidylcholine, unsaturated fatty acids, omega-3 fatty acids, and sphingomyelin than subjects with severe disease.

Yetukuri et al. [240]	Subjects from the *Fibrate Intervention and Event Lowering in Diabetes (FIELD)* substudy, whose changes in proteome and lipidome were evaluated after receiving (a) fenofibrate 200 mg daily or (b) placebo.	No difference was found in HDL-C levels between groups (*p* > 0.05). HDL from the fenofibrate group had lower LPC and higher sphingomyelin and Apo A-II.

## Abbreviations

ABCA-1: ATP-binding cassette transporter A-1
Apo A-I: Apolipoprotein A-I
CAD: Coronary artery disease
CE: Cholesteryl ester
CETP: Cholesteryl ester transfer protein
CVD: Cardiovascular disease
CVR: Cardiovascular risk
DM2: Type 2 diabetes mellitus
FC: Free cholesterol
HDL: High-Density Lipoprotein
IDL: Intermediate-Density Lipoprotein
LCAT: Lecithin-Cholesterol Acyltransferase
LDL: Low-Density Lipoprotein
LPC: Lysophosphatidylcholine
MPO: Myeloperoxidase
PC: Phosphatidylcholine
PLA_2_: Phospholipase A_2_
PON-1: Paraoxonase-1
RCT: Reverse cholesterol transport
SAA: Serum Amyloid A
SNP: Single-nucleotide polymorphism
SR-BI: Scavenger Receptor class B type I
sPLA_2_: Secretory Phospholipase A_2_
TAG: Triacylglycerides
VLDL: Very low-density lipoprotein.
